# Outer Brain Oscillations in Down Syndrome

**DOI:** 10.3389/fnsys.2019.00017

**Published:** 2019-05-07

**Authors:** Marcel Ruiz-Mejias

**Affiliations:** Systems Neuroscience, Institut d’Investigacions Biomèdiques August Pi i Sunyer, Barcelona, Spain

**Keywords:** down syndrome, oscillation, sleep, alpha, gamma, inhibition, mouse model, EEG

## Abstract

The present article reviews the relationship between sleep and oscillatory activity in Down Syndrome (DS), as well as the featuring emergent rhythmic activity across different brain states. A comprehensive discussion of the data from electroencephalographic studies in DS humans and transgenic/trisomic mouse models is provided, as well as data from signals collected from local field potentials (LFP) and intracellular recordings in DS mouse models. The first sections focus specially on the alpha phenotype consistently observed in DS subjects, as well as its description in DS childhood and aging. Subsequently, a review of the data reported in DS mouse models is presented with the aim to deepen on the mechanisms underlying altered rhythmic patterns. Further sections situate the state-of-the-art of the field, with a discussion on the possible circuit alterations that may underlie impaired alpha and gamma oscillatory activity. A further aim is to highlight the importance of studying network oscillatory activity in mouse models to infer alterations in the underlying circuits related to cognition, such as in intellectual disability. In this direction, a view of alpha and gamma rhythms generated by the cerebral cortex as a tool for evaluating an unbalance between excitation and inhibition in DS is claimed, which points out toward an over-inhibited network. A final aim is to situate oscillatory activity as a key phenomenon that may be used as a biomarker for monitoring as well the effect of novel therapeutic strategies.

## Introduction

Unraveling the alterations of the underlying circuits in neurobiological disorders is with no doubt one of the most interesting applications of studying brain oscillatory activity. Sleep and brain oscillatory activity in Down Syndrome (DS) is a particularly explored field of study, with early findings in sleep alterations in DS patients. Nowadays it is well accepted the relationship of sleep with cognitive function, due to its implication in processes such as memory consolidation. As well, rhythmic activity of neural networks such as alpha or gamma waves has been related widely to cognition in the last decades. Both sleep and oscillatory activity are tightly related, as the major oscillatory feature of sleep -the slow oscillation (∼1 Hz)- shows an exquisite interplay of different forms of rhythmic neuronal activity. Sleep and oscillatory activity in DS has been studied from the mid 20th century, but literature has appeared sporadically in the last decades. From the early descriptive studies, few works reported the differential features of sleep in DS individuals, showing a wide diversity but consistency of results. Some studies addressed network oscillatory activity emerging from external brain circuits of DS individuals, with results related to different frequency bands of the electroencephalogram (EEG) signal. Even fewer studies have been published in models, but interestingly, some of them focused in the network mechanisms that may explain intellectual disability and cognitive deficits observed in DS.

In the last 50 years, sleep and oscillatory activity in DS has been a focus of interest due to the evident alterations of sleep patterns in individuals with this chromosomal alteration, consisting on a partial or total trisomy of chromosome 21. The description of oscillatory activity during sleep and its functional mechanisms (Steriade et al., 1991; Contreras et al., 1996; McCormick and Bal, 1997; Chauvette et al., 2011) allowed the exploration of the emerging cortical rhythmic patterns associated with sleep stages, such as slow wave sleep. This type of activity has been proposed to be involved in main physiological and cognitive roles, such as synaptic homeostasis (Cirelli and Tononi, 2015), memory consolidation (Bigum et al., 1970; Buzsáki, 1998; Miyamoto et al., 2016), and even has been described as the default mode activity in the neocortex (Sanchez-Vives and Mattia, 2014). It is well studied that the slow oscillation orchestrates several types of rhythmic activity both during sleep and, interestingly, in some regimes of anesthesia (Steriade, 2006; Chauvette et al., 2011; Ruiz-Mejias et al., 2011), a fact that helped to study oscillatory patterns in animal models, thus allowing the possibility to study its alterations in DS models such as transgenic and trisomic mice.

Oscillatory activity is typically studied in the surface of the head by means of EEG recordings in humans. The first EEG recordings in DS individuals were performed by Gunnarson (1945) in the 40’s, concretely in DS children, by Vergani and Aldeghi (1958); Beley et al. (1959); Gibbs et al. (1965) in the 50’s, and later by Hirai and Izawa (1964) in the 60’s. Altered sleep patterns in DS humans were firstly observed by Petre-Quadens and Jouvet (1967); Castaldo (1969); Feinberg et al. (1969) in the 60’s by means of polysomnographic recordings. Another posterior study in the 70’s by Clausen et al. (1977) combined EEG recording with power spectrum analysis in different frequency bands to study oscillatory activity in DS subjects in different sleep stages. These studies showed consistently increased waking periods as well as reduced REM sleep in DS individuals. In later studies EEG signals were combined with localization of current sources, measures of cognitive performance, among others. Initial studies described as well how DS brain responded to external stimuli in form of evoked potentials in the 60’s and early 70’s (Barnet and Lodge, 1967; Bigum et al., 1970), while work in the 60’s with EEG recordings described as well the increased occurrence of epilepsy in DS (Borselli and Sferlazzo, 1963; Seppäläinen and Kivalo, 1967). Sleep patterns have found to be altered in DS children, with increased wakefulness, increased percentage of stage 1 of NREM sleep, and decreased REM sleep (Miano et al., 2008). The works in this field mostly characterized EEG patterns focusing in alterations in the alpha rhythms. The following are studies which relay on the interictal altered EEG sleep and oscillatory and patterns in DS individuals. Later, we review the work performed in mouse models of DS to foster explanations for the impairment observed in humans.

## Oscillations in DS Adults

The studies reviewed here commonly report alterations in the emergent oscillatory activity related to alpha oscillations. A firs evidence of this in sleeping/awake humans with DS performed power spectrum analysis –a transformation of the amplitude of the signal in the frequency domain- in EEG recordings to evaluate oscillatory activity across waking and sleep stages (Clausen et al., 1977). Clausen et al. (1977) reported increased power of frequencies between 1 and 20 Hz in all stages of sleep with exception to a narrow band of 8–13 Hz corresponding to alpha waves. The differences in power were more prominent in stages 1, REM sleep and the waking state. In addition, in the waking state, a peak in the alpha band in controls was not present in DS humans.

The alpha alteration background seems to correlate with cognitive performance and age. Reporting evidence on this statement, a study performed EEGs, psychometric testing, quantitative computed tomography, and positron emission tomography with fludeoxyglucose –that provides a measure of brain metabolism- in 19 young DS subjects, 9 older DS subjects and 13 healthy control subjects. When comparing the age-matched patients with normal alpha background subjects, older DS patients with decreased alpha background had dementia, fewer visuospatial skills, decreased attention span, larger third ventricles, as well as a global decrease in cerebral glucose utilization with parietal hypometabolism. Despite this, in the young patients with DS the EEG background did not correlate with psychometric or metabolic findings, but, however, in the neuroanatomical analysis the third ventricles were significantly larger in those with abnormal EEG background (Devinsky et al., 1990). Alpha alterations have been also measured as ratios between eyes-closed and eyes-open brain states. A study showed that alpha band power ratio between eyes-closed and eyes-open recording in the occipital area of waking EEG of DS humans was substantially increased compared to controls, thus showing he power of alpha waves was significantly lower in DS patients during the open-eyes state, with the degree of alterations correlating with neuropsychological test scores of DS subjects (Partanen et al., 1996). In DS adolescents, a lower power in the alpha waves was also reported, as well as in the beta and lower gamma EEG bands. As a novelty, DTF (directionality) values globally prevailed from right to left occipital areas in normal young subjects and in the opposite direction in DS patients (Babiloni et al., 2009).

In an effort to provide an improved spatial resolution of the previously observed alterations, further work aimed to identify the current sources of the alterations in wave activity. A second study in adults, Babiloni et al., 2009 combined EEG recording with LORETA technique (Low Resolution Electromagnetic Tomography), which allowed the correlation of EEG signals to the Current Source Density (CSD) representations in three dimensions. Central, parietal, occipital, and temporal cortical sources of resting alpha and beta rhythms were reported to be lower in amplitude in DS, whereas the opposite was true for occipital delta cortical sources. The authors argued that this result in alpha cortical source is consistent to what is typically found in Alzheimer’s disease, which is often associated to older DS humans (Babiloni et al., 2010). In this direction, further work combined also EEG with e/sLORETA technique (exact/standard LORETA) and provided correlation with cognitive performance. In similar frequency bands than the studies of Babiloni et al. (2010), they reported increased CSD in theta, alpha1 and beta1 classical bands and in individual alpha peak frequency-adjusted bands, while relative alpha2 was decreased. A negative correlation between cognitive performance and theta/alpha CSD in the right frontal lobe and right posterior cingulate cortex was also found (Velikova et al., 2011).

Despite EEG studies revealed valuable information, spatial and tissue filtering limitations of the technique motivated the exploration of magnetoencephalographic (MEG) signals in DS adults, which consistently found a decrease in alpha oscillatory activity. Data shows an attenuation of the “mu” rhythm in DS subjects –which refers to alpha oscillations related to sensorimotor areas- with a distinct laterality in the pattern of mu suppression, as well as distinct cortical pattern activation in the action observation network and asynchronous firing of activated areas. These results supported a dysfunction of the mirror system, where actions that are performed by others are recognized by activation of the same neural substrates that are involved in the actual execution of the observed action (Virji-Babul et al., 2008, 2010).

In summary, while waking EEG general oscillatory activity is increased in adolescent and young DS adults in parietal areas and beta and gamma is reduced in occipital areas, the studies show consistent absence of peaks and reductions in alpha rhythms in the neocortex of DS subjects. Sleeping DS subjects also show increased oscillatory activity in a wide range of frequencies across sleep stages in DS individuals, again with the exception of alpha oscillations. Despite these results, is worth to mention the difficulty of extracting high frequency components such as gamma oscillations from the EEG signals due to tissue filtering between neocortex and the recording electrodes, and the possible limitations in sampling frequency of acquiring signals from multiple channels, as well as that gamma frequencies are especially sensitive to normalization and noise artifacts. Table 1 summarizes the findings exposed in this section.

**Table 1:** Summary of findings of oscillatory activity in down syndrome (DS) adults.

First author of the work (year of publication)	DS subjects (recording technique)	Main findings in DS
Clausen et al., 1977	DS adolescents and young adults (EEG-polysomnography).	Increased oscillatory activity from 1 to 20 Hz during waking and sleep states with exception to alpha oscillations in parietal areas. Peak in alpha oscillations in waking state was not present.
Devinsky et al., 1990	Waking young and older DS adults (EEG).	Dementia, fewer visuospatial skills, decreased attention span, larger third ventricles, and a global decrease in cerebral glucose utilization with parietal hypometabolism in older DS adults with decreased alpha oscallations. Larger third ventricles were in young DS adults with decreased alpha oscillations.
Partanen et al., 1996	Waking DS adults with eyes closed and eyes open (EEG).	Increased alpha band power ratio between eyes-closed and eyes-open in occipital area in DS (decreased alpha oscillations in the eyes-open state). Correlation between the degree of changes and neuropsychological test scores.
Babiloni et al., 2009	Resting DS adolescents with eyes closed (EEG).	Reduced alpha, beta and gamma oscillations in occipital area. Directionality in occipital area prevailed left to right in DS (opposite in controls).
Babiloni et al., 2010	Resting DS adults with eyes closed (EEG-LORETA).	Lower current sources of alpha and beta current sources in central, parietal, occipital, and temporal cortical areas. Increased delta cortical sources in occipital area.
Velikova et al., 2011	Waking DS adults (EEG-e/s LORETA).	Increased current sources in theta, alpha1 and beta1 absolute bands (averaged electrodes). Reduced current sources in relative alpha2 band that positively correlated with cognitive tests (averaged electrodes). Increased current sources in alpha peak frequency-adjusted bands (alpha1, alpha2 and theta) in occipital areas. Negative correlation between cognitive performance and theta/alpha current source in right frontal lobe and right posterior cingulate cortex.
Virji-Babul et al., 2008, 2010	DS adults (MEG).	Reduced mu (alpha) rhythms in sensorimotor areas. Disruption of the action observation cortical network.


## Oscillations During Development and Aging in DS

One of the issues in the study of oscillatory activity in DS humans is its different consolidation during development, which found as well consistent alterations in alpha waves. An EEG study by Schmid et al. (1985) in the first years of life of DS children, they reported an increase in absolute power in theta oscillations in the fronto-central area, whereas alpha absolute power was slightly increased or even decreased. They found as well that the most prominent differences were when calculating the relative power of alpha oscillations, which was reduced starting at the age of 6 months, and that the reduction was more prominent with growth (Schmid et al., 1985). DS children have been reported as well to have some degree of lag of maturation of EEG-sleep patterns. In this direction, Hamaguchi et al., 1989 reported that, in addition to a low incidence of quiet sleep (non-REM) in the first 3 months, DS children had less spindles and later disappearance of frontal sharp waves, which are common during the first stages of development (Hamaguchi et al., 1989). When looking at the activity between area pairs in subjects ranging from 9 to 26 years old, it seems that coherence strongly and consistently discriminates between DS and control groups (McAlaster, 1992). A study in DS children provided evidence of decreased alpha waves during REM sleep in the occipital area, supporting differences during development in oscillatory activity of DS individuals, while beta, theta, and delta bands did not differ significantly between the groups (Śmigielska-Kuzia et al., 2005).

Parallel work in DS individuals explored the impairment of oscillatory activity in aging. When comparing DS individuals with non-DS with mental retardation and control subjects, occipital alpha-peak frequency was decreased with age in DS individuals. Furthermore, in older subjects this slowing was accompanied with an increase of activity in 6–8 Hz –theta oscillations, notice that 8 Hz is the limit between theta and alpha oscillations-, but didn’t correlate with cognitive performance in the daily life of individuals (Ono et al., 1992; Ono, 1993). Further evidence on this also supported a slowing in EEG oscillatory activity in the temporal area in DS aging adults aged 20 to 60. Concretely, they work showed that DS and Alzheimer’s disease (AD) patients had more prominent slow, delta and theta activity than control subjects. However, in this case those findings significantly correlated with and an age-related decline of cognitive functions in DS humans (Soininen et al., 1993). A slowing in frequency of oscillatory activity in EEG recordings was also reported in DS aging subjects, where the authors found significant differences between mean frequency of EEG power in delta, theta, alpha, and beta bands was 9.37 at 20’s in DS individuals, while in controls differences were non-significant between individuals in their 20’s and 60’s (Murata et al., 1994). The slowing in alpha rhythmic activity may be related also to the presence of Alzheimer-type dementia in DS individuals, as a follow-up study describes. The authors showed that the slowing of occipital alpha oscillations was related to a cognitive decline in a set of patients that were diagnosed post-mortem as presenting a severe form of AD (Visser et al., 1996). Further evidence supported the slowing in the dominant alpha peak in aging, also in a follow-up study in large population of DS subjects. Here, oscillations in resting EEGs from the frontal, central and occipital regions were examined in groups of subjects in intervals of 5 years. The number of subjects with DS who showed a dominant component within 8 Hz band of the basic rhythm reached maximum in its appearance rate at 40 to 44 years of age in the occipital area, while in healthy subjects the presence of this 8 Hz rhythm at this age was in a minority of cases. Confirming previous results, a slowing in this basic rhythm progressed already at 30 to 34 years in DS subjects. Opposite to this, in non-DS mental retardation, the number of subjects who showed dominant component at 8 Hz reached maximum at 45 to 49 years of age, and this slowing of the basic rhythm was not as clear as in DS. In a parallel follow-up study published in the same article, EEGs were recorded repeatedly once a year during 8 or 9 years in persons with DS and with non-DS mental retardation. The result was that although the lowering in EEG frequency to 8 Hz took place in various years of age individually, earlier distinct decrease of the frequency was commonly noticed in DS subjects (Katada et al., 2000). As stated, in this last two studies the authors hypothesized that the earlier slowing could be a senile sign and be related to the decline of brain function referring to AD. Supporting this idea, decreases in theta oscillations in several regions of the neocortex negatively correlated with cognitive impairment, and could discriminate cognitive decline in DS adults with AD (Salem et al., 2015). A summary of the findings in this section is available in Table 2.

**Table 2:** Summary of findings oscillatory activity in DS during development and aging in DS.

First author of the work (year of publication)	DS subjects (recording technique)	Main findings
Schmid et al., 1985	DS children (EEG).	Increase in absolute power of theta oscillations in fronto-central area. Slight increase or even decrease in absolute power of alpha oscillations. Decrease in relative power of alpha oscillations.
Hamaguchi et al., 1989	DS children (EEG-polysomnography).	Lag of maturation of EEG-sleep patterns. Less spindles and later disappearance of frontal sharp waves.
McAlaster, 1992	9 month to 26 year-old DS humans (EEG).	Coherence between pairs of areas strongly and consistently discriminated between DS and control groups.
Śmigielska-Kuzia et al., 2005	Sleeping DS children (EEG-polysomnography).	Decreased alpha waves during REM sleep in occipital area.
Ono et al., 1992; Ono, 1993	Waking DS adults across aging (EEG).	Occipital alpha-peak waves decreased in frequency with age in DS individuals. In older subjects slowing was accompanied with increase of activity in 6–8 Hz, but didn’t correlate with cognitive performance.
Soininen et al., 1993	Waking DS adults (EEG).	Slowing in EEG oscillatory (slow, delta, theta) activity in temporal area that correlates with cognitive performance and an age-related decline of cognitive functions in DS humans.
Murata et al., 1994	Waking DS adults (EEG).	Slowing in mean EEG oscillatory activity with aging.
Visser et al., 1996	Waking DS adults, follow-up study (EEG).	Slowing in occipital alpha oscillations oscillatory activity related to cognitive decline with aging and to incidence of Alzheimer’s disease.
Katada et al., 2000	Large population of waking DS adults including a follow-up study (EEG).	Earlier steep slowing of EEG alpha-peak in DS in frontal to occipital areas.
Salem et al., 2015	DS-AD adults (EEG).	Decrease in theta oscillations in several areas that negative correlate with cognitive performance that can detect cognitive deterioration.


## Sleep and Oscillations in DS Mouse Models

In parallel to the previously mentioned work in humans, EEG signals have also been obtained in several mouse models of DS. The analysis of neuronal oscillatory activity in DS mouse models started of course in the 2000’s with the irruption of transgenesis and related technologies. Since then, EEG-sleep patterns have been studied in a few of those models. In a first study in two transgenic mice, hSOD and hAPP -overexpressing the human genes SOD1 and APP, respectively, Colas et al., 2004 found that the number of REM sleep episodes were decreased and REM latency increased after lights off in hSOD mice, similarly to some sleep features in DS humans. In contrast, hAPP mice exhibited no change in REM sleep but an increase in waking and a decrease in slow wave sleep before light transition as well as an increase in theta power in REM and waking, also found in DS humans. In addition, sleep deprivation affected differently both models: while hSOD mice did not experience slow wave sleep or REM sleep rebounds after sleep deprivation but EEG activity in the delta-SWS activity was enhanced, hAPP mice exhibited slow wave sleep and REM sleep rebounds as well as enhancement of delta-slow wave activity (Colas et al., 2004). Similar features were studied in the same lab in trisomic models – Ts65Dn and TsjCe trisomic mice carrying different lengths, respectively, of extra chromosomic fragments homologous to regions in human chromosome 21. They found increased waking periods at the expense of non-REM sleep, increased power in theta waves during sleep and a delayed sleep rebound after sleep deprivation in Ts65Dn mice. In contrast, Ts1Cje had limited sleep and EEG abnormalities, showing only a delayed sleep rebound after sleep deprivation and no difference in the power of theta oscillations (Colas et al., 2008). As a hypothesis extracted from those studies, where theta oscillations were altered in hAPP and Ts65Dn mice, the authors suggested a possible correlation between APP over-expression and changes in hippocampal theta oscillations. A recent work provided evidence of altered sleep patterns in the trisomic Dp16 model, where increased waking time at the expense of NREM sleep was found. Despite alterations in oscillatory activity did not recapitulate the alpha phenotype observed in DS humans, EEG recordings showed theta, beta, and alpha abnormalities across different brain states, suggesting also impairment in the circuits (Levenga et al., 2018).

Undoubtedly, an interesting feature of mouse models is the possibility to underpin the mechanisms underlying the observed alterations. In this direction, slow wave activity was recorded in slices of the somatosensory cortex of Ts65Dn mice to elucidate the mechanisms behind sensory deficits in DS (Sutton, 2005; Bruni et al., 2010). In a characterization of the intrinsic and network properties of layer 4 regular spiking pyramidal neurons in the somatosensory cortex, spontaneous excitatory and inhibitory synaptic inputs to this cell type were found to be reduced, as well as the duration of neuronal UP states -i.e., active states in the slow oscillation that alternate with silent or DOWN states at ∼1 Hz. Importantly, the authors provide results of intracellular recordings that show a decreased intrinsic excitability of the layer 4 regular spiking cells, which explains the reduced synaptic activity and the shorter duration of UP states (Cramer et al., 2015). These findings contribute to the idea that cortical network in DS may be unbalanced, or at least less excitable due to intrinsic properties of neurons, as this work shows.

We performed a multidisciplinary work that studied oscillatory activity in the transgenic DS model TgDyrk1A. This transgenic mouse overexpresses DYRK1A, a double-kinase protein implicated in neurodevelopmental and synaptic processes and a candidate to generate cognitive deficits in DS. Alterations of oscillatory activity were studied in the prefrontal cortex *in vivo* with Local Field Potential (LFP, an EEG in depth) recordings. Neurons’ firing rate as well as gamma -30 to 90 Hz- oscillations were found to be reduced during the UP states of slow oscillations in the transgenic mice, which was proven both in waking and anesthetized mice, and providing further evidence in direction of a decreased cortical excitability. Propagation of UP states along the motor cortex was also slowed as they moved away from their site of generation in prefrontal cortex. These results made us hypothesize that the prefrontal cortex of TgDyrk1A is over-inhibited, which was tested by anatomical experiments. Excitatory and inhibitory synaptic contacts to both pyramidal –excitatory- and parvalbumin –inhibitory- neurons were counted, showing that inhibitory contacts were reduced specifically over inhibitory cells. This supported the idea of an effective “super inhibition” of the network, which was tested in a computational model that reported the structural changes were sufficient to explain the observed functional alterations. The findings pointed out to a reduction in recurrent inhibition as a mechanism that may explain cognitive deficits in DS (Ruiz-Mejias et al., 2016). A summary of the findings in DS mouse models is presented in Table 3.

**Table 3:** Summary of findings of oscillatory activity in DS mouse models.

First author of the work	Year of publication	DS subjects (recording technique)	Main findings
Colas et al., 2004	2004	SOD1 and hAPP mice (EEG).	Increase in waking and a decrease in slow wave sleep before light transition as well as an increase in theta power in REM and waking.
Colas et al., 2008	2008	Ts1Cje and Ts65Dn mice (EEG).	Increased waking periods at the expense of non-REM sleep in Ts65Dn mice. Increased power in theta oscillations during sleep and a delayed sleep rebound after sleep deprivation in Ts65Dn mice. Limited sleep and EEG abnormalities, delayed sleep rebound after sleep deprivation in TsCje mice.
Cramer et al., 2015	2015	Ts65Dn mice slices (intracellular).	Reduced spontaneous excitatory and inhibitory synaptic inputs to somatosensory cortex L4 regular spiking pyramidal neurons. Reduced UP state duration in these cells. Decreased intrinsic excitability in these cells.
Ruiz-Mejias et al., 2016	2016	TgDyrk1A mice (LFP).	Decreased firing rates and gamma oscillations in prefrontal cortex of anesthetized and waking mice. Slower propagation of UP states in frontal cortex of anesthetized mice. Reduced inhibitory contacts to inhibitory cells.
Levenga et al., 2018	2018	Dp(16) 1Yey/+ mice (EEG).	Theta decreased –within the light phase- and beta increased in waking. Alpha and beta waves increased and delta decreased in NREM sleep. Beta increased in REM sleep. Increased time awakke at the expense of NREM sleep.


## Where Do We Come From?

This paper reviews the literature on interictal EEG sleep and oscillatory activity in DS individuals, gathering as well the findings in this field on DS mouse models. Previous studies characterized well the sleep activity in DS, including those episodes related with epileptic activity. As well, EEG studies provided first insights of oscillatory activity in DS humans both during sleep and wake periods, and also associated to the development and aging life periods. These works, conversely, were constraint by limitations in spatial and temporal resolution, due to the fact of being non-invasive techniques and the technical development in those years, even the LORETA techniques approach provided spatial localization of EEG current sources in outer brain areas.

The field of sleep and oscillations in DS is diverse, and as stated above, literature has appeared dispersedly during the last decades, showing widespread results in humans and mouse models. Since the early polysomnographic studies a remarkable leap has been carried out from the main observational studies in humans, and this review shows there is a wide and open field of study for research in this topic. The works presented here point out to unbalanced network excitation and inhibition in underlying cortical areas in DS, which generate aberrant rhythmic activity at different levels.

Brain architecture alterations have been also characterized DS humans (Crome et al., 1966; Weis et al., 1991; Pinter et al., 2001) as well as in trisomic DS models (Aldridge et al., 2007). Given that the origin of EEG/LFP signals comes from both structural and synaptic components (Buzsáki et al., 2012), the observed phenotypes in oscillatory patterns both in DS humans and models may have also a structural component comprising the quantity and packing of all neuronal compartments in the local circuit, including synapses.

## Where Do We Go?

Our lab work demonstrates that oscillatory activity, understood as an emergent property from the outer networks of the brain, is useful to infer alterations in the underlying circuits. Moreover, novel biomarkers may be obtained from the study of this activity, that might be used to track the effect of new therapeutic approaches, both at basic and clinical level. At this level, an alpha rhythm decrease in DS individuals as well as a slowing of a dominant alpha peak in aging DS subjects seem to be consistent findings across studies that could be used with this purpose. Further works can also take a closer look at the communication between cortical areas with means of cross-correlation and coherence analysis of network signals, or increase the spatial resolution and improve tissue filtering effects of brain surface recordings with means of MEG techniques to extract gamma rhythms related to cognition (Rojas et al., 2008; Buzsáki and Wang, 2012; Siegel et al., 2012). EEG recordings –and video-EEG- are critical to diagnosis and monitor alterations in neurological processes such as epilepsy (Aldrich and Jahnke, 1991; Cascino, 2002; Staba et al., 2014) or useful to early characterize dementia or AD (Al-Qazzaz et al., 2014; Houmani et al., 2018). A further use may be the inclusion of EEG/MEG recordings in clinical trials to track the effect of novel therapeutic strategies in DS, such as the green tea polyphenol and specific DYRK1A inhibitor epigallocatechin-3-gallate (De la Torre et al., 2014; Stagni et al., 2017) Importantly, EEG and LFP recordings in mice will provide valuable information of oscillatory activity regarding aneuploidy mimicking DS, as well as the underlying circuit impairment that explain the observed emerging alterations. The fostering of new data in DS brains will improve the knowledge of how brain works in intellectual disability to eventually enable a better quality of life for DS patients.

## Alpha and Gamma Rhythms as Potential Biomarkers of Cortical Inhibition Impairment

It has been shown that event-related synchronization (ERS) in the alpha band has a functional correlate of inhibition in cognitive, motor or auditory tasks (Klimesch et al., 2007; Jensen and Mazaheri, 2010; Strauß et al., 2014). Despite this, there is few knowledge of which would be the underlying circuit mechanisms supporting this process. In this review we showed how changes in oscillatory activity could serve as a biomarker of alterations in the underlying circuit. To the date, the neurophysiological mechanisms responsible of alpha rhythms are still unclear, although GABAergic feedback from interneurons has been proposed as a participating mechanism in the generation of these oscillations (Jones et al., 2000; Lõrincz et al., 2009). Here we show a consistent decrease and slowing in alpha waves in DS. The pending questions are whether these alterations in oscillatory activity are related to changes in the effective functional inhibition, and also which is the alteration in the cortical circuitry that is supporting impairment in oscillatory activity. A monoaminergic unbalance –i.e., noradrenaline and serotonine- has been also described in aging DS humans (Dekker et al., 2017), which may be reated to the observed slowing of the EEG oscillatory activity. In turn, there are consistent evidences that the GABAergic system is responsible of generating gamma oscillations (Cardin et al., 2009; Buzsáki and Wang, 2012; Siegel et al., 2012). The precise timing of the synaptic activity of this system, the structural synaptic dispositions and the interplay with resonance generators at cellular level in generating either alpha and gamma oscillatory activity remains elusive, and further research will be needed to elucidate the putative dynamical capacity of inhibitory circuitry to participate in both kinds of activity, with emphasis in alpha oscillations.

## Conclusion

This article reviews the literature on the relationship of sleep ant oscillatory activity in DS, coming from activity from the outer areas of the brain. A consistent phenotype has been observed regarding alterations that down-regulate alpha oscillations in different brain states, with a general up-regulation of EEG oscillatory activity in sleep. During brain circuits’ development across childhood, DS children show a lag of maturation of rhythmic patterns with an uneven settlement and expression of alpha waves. In turn, the dominant alpha rhythm of the EEG experiences a slowing from adulthood and across lifespan, with may be related in aging DS humans to dementia. This review also claims mouse models are valuable tools for characterizing the network mechanisms behind intellectual disability observed in DS humans. Results are presented from studies that try to go a step beyond the observation and provide an explanation for the alterations observed at network level, thus demonstrating a decrease in neuronal activity in DS neocortex. These alterations are present either at intrinsic or synaptic levels, which are not exclusive. In addition to the early work focused on alpha oscillations, a deeper understanding of further oscillatory patterns is claimed here, such as gamma oscillations, which are directly related to cognitive function. Research work also provides evidence that functional and anatomical synaptic studies in transgenic/trisomic models may help to determine the underlying mechanisms of cognitive dysfunction in DS. Future work might cover the gaps to determine the relationship between intrinsic and synaptic neuronal properties and microcircuit contributions in the generation of altered network oscillatory patterns implicated in cognition in DS models, and clarify whether alterations in the neocortex are ubiquitous in the whole cortex or rather area specific.

## Author Contributions

MR-M wrote the manuscript.

## Conflict of Interest Statement

The author declares that the research was conducted in the absence of any commercial or financial relationships that could be construed as a potential conflict of interest.
